# Sensory and Chemical Characterization of Upcycled Pomace- and Whey-Based Piquette Beverages

**DOI:** 10.3390/foods14183240

**Published:** 2025-09-18

**Authors:** Dean G. Hauser, Rahul Sen, Scott R. Lafontaine, Chris Gerling, Luann M. Preston-Wisley, Timothy A. Demarsh, Samuel D. Alcaine

**Affiliations:** 1 Department of Food Science, Cornell University, Ithaca, NY 14853, USA; cjg9@cornell.edu (C.G.); lmp3@cornell.edu (L.M.P.-W.); tad24@cornell.edu (T.A.D.); alcaine@cornell.edu (S.D.A.); 2 Department of Food Science, University of Arkansas, Fayetteville, AR 72704, USA; rsen@uark.edu (R.S.); scottla@uark.edu (S.R.L.)

**Keywords:** upcycled food, yogurt acid whey, grape pomace, piquette, descriptive analysis, sustainability, fermentation

## Abstract

Upcycling, or utilizing materials that would otherwise go to waste, enables the creation of novel products that offer sustainability advantages and generate additional value. This study evaluates the feasibility of producing alcoholic beverages using yogurt acid whey (YAW) and grape pomace (GP), byproducts of the dairy and wine industries, respectively, and compares them to commercial grape pomace beverages (piquettes) in terms of sensory attributes and chemical composition. Two YAW-GP piquettes were produced, and five commercial piquettes were obtained. Sugars and organic acids were quantified using HPLC-RID, and semi-quantitative volatile composition was determined using HS-SPME-GC-MS/MS. Descriptive analysis was conducted using a trained panel of 11 individuals. The YAW products had higher ratings for dairy, salty, acidic, and umami attributes, and lower ratings for bitterness, sweetness, red fruit, dried fruit, and overall fruity characteristics. YAW beverages were higher in titratable acidity (TA), lactose, lactic acid, citric acid, galactose, hexanoic acid, 3-methylpentanol, 1-octanol, and 1-octen-3-ol, and lower in ethanol and linalool. The commercial products were differentiated based on ethanol content, red fruit, dried fruit, fruitiness, chemical, and barnyard aromas. These results can be used to understand the breadth of chemical and organoleptic signatures of this new beverage category, which can be leveraged by stakeholders interested in entering the market.

## 1. Introduction

Food waste and loss across the supply chain carry major environmental burdens and are estimated to have accounted for 8–10% of total anthropogenic greenhouse gas emissions between 2010 and 2016 [1]. In recognition of the scale of food loss and waste prior to retail, the wasted food scale designed by the United States Environmental Protection Agency indicates that beyond preventing wasted food (source reduction), upcycling (converting food waste into new food products), and donation offer the most benefits to the environment, highlighting a major need for research and development to support the upcycled food industry [2]. In addition to environmental benefits, upcycled food solutions can create significant economic and social benefits. According to Refed, upcycled foods have the potential to divert 1.94 million tons of food waste, prevent 5.56 million tons of carbon dioxide-equivalent emissions, and save 557 billion gallons of water, while simultaneously generating a net financial benefit of $2.8 billion, and creating 2950 jobs annually [3].

Grape pomace, the seeds, stems, and skins left over after pressing sweet white wine grapes or fermented red wine, is the main byproduct of winemaking, and is generated at a scale of roughly 15–16 kg of pomace for every 100 L of finished product [4,5]. In 2024, 761 million gallons of wine were produced in the US, resulting in roughly 432 million kilograms of pomace [6]. This material is rich in fiber, polyphenolic compounds, colorants, and minerals, which make it attractive for use in upcycled food products; however, the high moisture content and compositional variation in pomace due to grape cultivar and/or vinification techniques pose challenges in terms of its re-use and microbial stability, especially in applications where it must be stored for some period before use [7]. As shown in the meta-analysis performed by Antonic et al. [7], numerous studies have been conducted on the nutritional and organoleptic impacts of utilizing grape pomace for the fortification of plant-based foods (bakery items, purees), meat and fish products, and dairy products.

Beyond fortification of food products, grape pomace was historically used to produce beverages such as lora in classical Rome and piquettes in France [8,9]. These lower-alcohol (4–8% *v*/*v*) wine-like sparkling beverages were reportedly consumed by vineyard workers and were produced by adding water and/or juice as well as other fermentable substrates to grape pomace to referment [8,10]. More recently, piquettes have been gaining popularity among winemakers and consumers in the United States, Australia, and New Zealand, presenting a new low-cost product offering for winemakers which may allow for better engagement with younger consumers or those who are not wine drinkers [9,11].

Harutyunyan et al. produced piquette beverages from Muscat of Alexandria and conducted consumer testing which identified specific consumer segments who liked the products [8,12]. Watrelot and Hollis produced piquettes with Petite Pearl and Marquette pomace supplemented with cane sugar with different yeast strains and different water/pomace ratios and conducted a sensory study with an untrained sensory panel; the results indicated that the Petite Pearl-based beverages were preferred, and that pomace/water ratios and yeast type affected chemical composition and consumer preference [10]. Aside from these two investigations, there is a lack of literature on the production or characteristics of piquette beverages.

Whey is an underutilized liquid byproduct of cheese, yogurt, and other dairy foods manufacturing processes and has an estimated annual production rate of over 200 million tons globally [13]. Currently, about 50% of the whey produced in the United States and Europe is processed into protein concentrates, lactose, and other products for human food or animal feed, with the balance managed through waste treatment, application to agricultural land as fertilizer, or use as livestock feed [13,14,15]. Waste treatment costs are high for these effluents due to their biochemical oxygen demand, and processing costs to convert whey into powders or other streams can be prohibitive to smaller operations, pointing to a need for low-overhead upcycling solutions [13,15,16].

Whey and whey/milk permeate streams are an attractive base for upcycling in the form of fermented alcoholic and non-alcoholic beverages, sports drinks, and other upcycled food products that enable the addition of value [15,17,18,19], and several studies have investigated the production of whey beverages or spirits [20,21,22,23,24,25,26,27,28,29]. In the context of beer production, Pasta et al. utilized ricotta whey, or scotta, to produce novel upcycled products that fit existing beer style profiles [30]. Outside of alcoholic beverages, Thibodeau et al. developed a kombucha culture or symbiotic community of yeasts and bacteria (SCOBY) that is able to utilize lactose, potentially enabling the use of whey effluents as ingredients in novel kombucha beverages [31]. YAW in particular is a byproduct of the production of strained yogurts such as Greek yogurt, and typically contains roughly 3.33–3.5% lactose, 0.64–0.75% ash, and 0.17–0.37% protein and has a pH of 4.21–4.48 [16]. Strains of *Saccharomyces cerevisiae*, the most widely used yeast in the production of alcoholic beverages, are unable to utilize lactose directly as a carbon source, but the addition of exogenous lactase (β-galactosidase) enzymes allows for the hydrolysis of lactose into glucose and galactose, which can be efficiently converted to ethanol by most strains (galactose utilization efficiency can vary) [32,33].

Therefore, given the considerable quantities of YAW and GP produced every year, the recent revival of piquette beverages, and the need for actionable low-cost upcycling opportunities for dairy and wine processors, the main goal of this study was to create examples of YAW-based piquettes, and to compare the sensory profiles and compositions of these beverages to commercially available non-dairy piquette beverages. Specifically, this work both explores the potential for YAW piquettes as a new beverage style and benchmarks them against existing piquette beverages to understand where they may conform to or depart from commercially available products. This study is also the first to provide insights into the range of sensory and chemical profiles of commercial piquette beverages currently on market. Overall, this study highlights a new prospect using upcycled GP and YAW in beverage innovation to increase sustainability and reduce waste in both alcoholic beverage production and dairy processing.

## 2. Materials and Methods

### 2.1. Commercially Available Piquettes

Commercially available piquettes were obtained via donation or were purchased from a local wine shop (Table 1). Whey piquettes were produced at Cornell Agritech in Geneva, NY, USA. Production practices for the whey piquettes can be found in Section 2.2.

According to the vendors and/or producers of the various commercial piquette products, the production practices are as follows. For CF-W1, water was added to the pomace, soaked for 1–2 weeks, and pressed out. Some of the original wine was blended into the product, which was then bottle-conditioned. For CF-W2, water and cane sugar were added to pomace collected from a dry rose wine. For RB-C, Golden Russet, Ben Davis, and Idared juices were aged on red grape skins for six weeks. For PN-WJ, water and pinot gris juice were added to pomace. For RB-WJ, water was added to pressed skins of a still rose, fermented for 9 days, pressed again, blended with roughly 50% volume of unfermented juice, and bottled.

### 2.2. Whey Piquette Production

Two yogurt acid whey (YAW)-based piquettes were prepared for analysis in this study. One was prepared by fermenting pasteurized, carbon-filtered whey on Cabernet Franc grape pomace, and the other by aging a pre-fermented alcoholic acid whey beverage base on Cabernet Franc grape pomace. Fresh YAW was obtained from HP Hood in Vernon, NY and transported to the Food Science Department at Cornell University in Ithaca, NY in sanitized (StarSan, Five Star Chemicals, Arvada, CO, USA) 5-gallon food grade HDPE buckets and subsequently stored at 4 °C overnight until pasteurization. Pasteurization was carried out using a Brewzilla 3.1 (Kegland, Victoria, Australia) and involved holding the YAW at 75 °C for 20 min [34]. After pasteurization, the YAW was brought back to room temperature using a stainless steel immersion chiller and was stored at 4 °C overnight until filtration. Three-stage filtration was carried out using a Colombo Inox plate-and-frame filtration system (Fermtech Ltd., Kitchener, ON, Canada) with Fermtech filter pads utilized in the following order: #1 coarse, #2 polishing, and #3 sterile filtration (Fermtech Ltd., Kitchener, ON, Canada). After the final filtration, carbon filtration was carried out using carbon filter sheets (PRO3ACN, Carlson Filtration, Barnoldswick, England, U.K.), and treated YAW was stored at 4 °C in sanitized 5-gallon food grade HDPE buckets until it was brought to Cornell Agritech in Geneva, NY, where it was frozen until its use in the fermentation trial. Pre-fermented YAW alcoholic beverage base was obtained from a local beverage producer and stored at 4 °C in sanitized 5-gallon food grade HDPE buckets until use. Cabernet Franc grape pomace was obtained from a local winery during the pressing of a dry Cabernet Franc wine of the 2022 vintage and was stored frozen until use in fermentation and aging trials.

For the fermentation trials, the treated whey and whey beverage bases were removed from cold storage to thaw and attemperate, and the former was subsequently dosed with Maxilact A4 (DSM-Firmenich, Maastricht, The Netherlands) at a rate of 40 ALU/g lactose. Potassium metabisulfite (Presque Isle Wine Cellars, North East, PA, USA) was added to the whey targeting 50 ppm sulfur dioxide, and the pH was adjusted to 3.5 using tartaric acid. A total of 24 h after sulfur treatment, the whey was inoculated with 1 g/gallon of LALVIN EC1118™ (Scott Laboratories, Petaluma, CA, USA), which had been rehydrated with GO-FERM PROTECT EVOLUTION™ (Scott Laboratories, CA, USA), and was supplemented with 0.25 g/L FERMAID K™ and 0.5 g/L DAP (Scott Laboratories, CA, USA). The inoculated whey or whey beverage base was then mixed with the thawed grape pomace, at a ratio of 20 kg of whey to 10 kg of grape pomace, in 15-gallon stainless steel tanks. Fermentation or soaking was carried out for 6 days, after which pomace was separated from the products and the beverages were transferred into 3-gallon glass carboys and stored at 4 °C until their transport to Cornell University in Ithaca, NY, for kegging. Upon arrival in Ithaca, the beverages were transferred into sanitized 5-gallon ball-lock kegs in which they were stored at 12 PSI CO_2_ overpressure. After carbonation, kegged YAW piquettes and bottled piquettes were transferred to 12 oz amber glass bottles and sealed with crown caps until sensory analysis or chemical analysis.

### 2.3. Descriptive Sensory Analysis

Descriptive analysis was carried out on the seven piquette beverages described in Section 2.1 and Section 2.2, following a protocol that was approved by the Cornell University Institutional Review Board prior to the start of data collection (protocol # IRB0147351). Panelists were recruited from a voluntary departmental graduate student panel which had been previously screened for sensory acuity, trained on scaling basic tastes, and had also been utilized previously for several descriptive analysis studies of beverages including wine and tequila. Panelists were compensated through the departmental panel for participation in this study. In total, 11 panelists participated in this study. All panelists provided informed consent and indicated that they were 21 or older; did not have allergies or sensitivities to alcohol, dairy, apples, or sulfites; were not pregnant; and were also asked not to eat, drink, or smoke for one hour prior to training or evaluation sessions. Panelists’ ages ranged from 25 to 32 years of age with a median age of 27; 6 of the panelists identified as male and 5 as female.

For the basic taste training, panelists scaled intensities of aqueous solutions and suspensions of sweet (sucrose), bitter (quinine), astringent (alum), acidic (tartaric acid), and umami (monosodium glutamate). During the wine and tequila panels, panelists were trained on an array of aroma and flavor attributes in addition to mouthfeel attributes of body and alcohol strength. For the present study, aroma references included standards from the Le Nez Du Vin Masterkit (See Table 2) (Editions Jean Lenoir, Cassis, France). For a barnyard reference, a *Brettanomyces claussenii* (OYL-201, obtained from Omega Yeast Labs, Chicago, IL, USA) culture was prepared in reconstituted dry malt extract, and mixed 1:1 with the CF-W2 reference. For a dairy reference, Greek yogurt (10 g/L) was mixed with the CF-W1 reference.

The study consisted of one language generation session, followed by three training sessions and two evaluation sessions. On the day of each individual session, an appropriate number of 12oz bottles of each sample were removed from refrigerated storage and poured into 12-ounce black polystyrene cups (Crown Display Inc., Pittston, PA, USA) in 1-ounce aliquots. Bottles were removed from refrigeration and samples were poured one hour in advance, to allow them to come to room temperature and for some of the carbonation to dissipate. All cups were labeled with randomly generated three-digit blind codes which were unique for each session and sample, except for reference samples which were labeled as such. The RedJade sensory program (RedJade Software Solutions, LLC, Redwood City, CA, USA) was used for all data collection, generation of blind codes, and determination of serving order during training and data collection. During the language session, a subset (n = 7) of panelists provided initial feedback and discussion regarding the samples, and generated descriptors which were used as a basis scaling exercises. During the first training session, the panelists were presented with a subset of the samples and asked to smell the samples and scale the orthonasal aroma intensities of several attributes, followed by tasting and expectorating the sample and rating retronasal aroma intensities, followed by tasting and expectorating the sample again to scale basic taste and mouthfeel attributes. After the data was collected, a discussion was facilitated which focused on determining which attributes best delineated the differences between the samples and reaching consensus intensity ratings for these selected attributes. In sessions two and three, panelists were again presented with a range of products and asked to complete intensity scaling exercises; after discussion, the participants agreed that there were minimal differences between the ortho- and retronasal aromas of the products in terms of their attributes and intensities, so evaluation sessions only included the retronasal modality. Evaluation sessions consisted of panelists tasting and expectorating each sample and rating retronasal aroma intensities, followed by tasting and expectorating the sample again to scale basic taste and mouthfeel attributes, all on a structured line scale of 0–15 points, where 0 was anchored by “none”, 2 was anchored by “just detectable”, 5 was anchored by “slight”, 10 was anchored by “moderate”, and 15 was anchored by “extremely high”.

### 2.4. Analytical Methods

Volatile compound screening was performed on duplicate 1 mL piquette samples thawed at room temperature in a 10 mL amber screw-cap vial (containing 0.4 ± 0.01 g of NaCl). Untargeted/qualitative SPME GC-MS/MS approach was used to screen the volatile composition of the different piquette sample in duplicate using a Shimadzu Nexis GC-2030 (Shimadzu, Kyoto, Japan) system equipped with a GCMS-TQ8050NX triple-quadruple mass selective detector and AOC-6000 Plus autosampler (Shimadzu, Kyoto, Japan). The volatiles were adsorbed on a 1 cm-long divinylbenzene/carboxen/polydimethylsiloxane (DVB/CAR/PDMS) solid-phase micro-extraction (SPME) fiber (Supelco, Bellefonte, PA, USA). The capillary column used for separation and characterization was ZB-5MSplus (30 m × 0.25 mm × 0.25 µm) (Phenomenex, Torrance, CA, USA). Samples were incubated and extracted at 50 °C for 10 min. The injection was performed in spitless mode with 3 min desorption time at an inlet temperature of 240 °C. Helium was used as the carrier gas at a flow rate of 1 mL/min and a constant inlet pressure of 46.7 kPa. The initial oven temperature of 35 °C was held for 5 min, then increased to 150 °C at a rate of 5 °C /min. Subsequently, in the final ramp, the temperature was raised at a rate of 8 °C /min to 280 °C and held for 5 min, resulting in a total run time of 49.25 min.

Untargeted/qualitative analysis was performed using the methods utilized by Maust et al. and Sen et al. with slight modifications [35,36]. In brief, the mass spectrometer (MS) was operated in full scan mode (40–400 *m*/*z*) with ion source temperatures of 240 °C and MS interface temperature of 290 °C. Compounds were identified with similarity rates of ≥90% and ±10 RI allowance using Shimadzu LabSolutions software, based on mass spectral libraries NIST2020 (National Institute of Standards and Technology, Gaithersburg, MD, USA) and Flavors and Fragrances of Natural and Synthetic Compounds (FFNSC3, John Wiley & Sons, Inc., Hoboken, NJ, USA). A linear retention index was created using an alkane standard mix solution (C6–C20) to further confirm the RI of the molecule. An amount of 10 µL of deuterated hexanal-d12 (1 µg/uL) was added as an internal standard (IS) to each sample vial to avoid the interference with naturally occurring compounds, reduce peak coelution, and ensure accurate quantification. Also, at the beginning and end of each sample set, in order to check the consistency of the method, an analytical standard solution mix containing a known amount of 101 volatile compounds (representing different volatile classes) including hexanal-d12 as IS was analyzed (Table S1). These check samples were processed in a similar way to the piquette samples to check the variation in RI value, library identification, and amount of the compounds. A variation exceeding ± 20% in any of these criteria was flagged, prompting the preparation of a fresh solution, reprocessing, and tuning of the system. Concentration of the volatile compounds present were calculated semi-quantitatively with reference to the area of IS used as in Equation (1):

Analyte concentration (μg/mL) = (peak area of analyte)/(peak area of internal standard (IS)) × IS concentration (μg/μL) × IS volume (μL) × (1/sample volume (mL))(1)

HPLC analysis for sugars and organic acids was conducted by the Cornell Craft Beverage Analytical Laboratory (CCBAL; Cornell Agritech, Geneva, NY, USA). Samples were analyzed using a Prominence HPLC system (Shimadzu, Kyoto, Japan) equipped with a 300 × 7.8 mm Rezex™ ROA-Organic Acid H+ Column (Phenomenex, Torrance, CA, USA); a Photodiode Array Detector, model SPD-M20A (Shimadzu, Kyoto, Japan); and a Refractive Index Detector, model RID-10A (Shimadzu, Kyoto, Japan). A mobile solution of 0.005 N H_2_SO_4_ was used, and 20 μL of sample was injected. Duplicate injections were conducted for each sample and mean values were reported by CCBAL. Mobile phase was run at a constant rate of 0.5 mL/min for a 35 min run time at a column temperature and RID temperature of 45 °C. Prior to analysis, samples were diluted with an equal volume of HPLC grade water and filtered through a 0.22 μm 13 mm diameter PES filter. Quantification was carried out relative to standard curves which were constructed for each analyte; concentrations of 0.05, 0.1, 0.5, 1.0, 5.0, and 10 g/L were used for each analyte. pH was measured in technical duplicate using an iCinac analyzer (AMS Alliance, Rome, Italy) and titratable acidity was measured in technical duplicate using a Hanna Autotitrator (Hanna Instruments, Smithfield, RI, USA).

### 2.5. Statistical Methods

Panel analysis, mixed-model analysis of variance (ANOVA), Tukey HSD post hoc means comparisons, principal component analysis (PCA), multiple factor analysis (MFA), and Pearson correlation analysis were carried out using XLSTAT-sensory (Addinsoft, Paris, France). Heatmaps were generated using JMP Pro 17 (SAS Institute Inc., Cary, NC, USA).

## 3. Results and Discussion

### 3.1. Sensory Analysis

**Figure 1:**
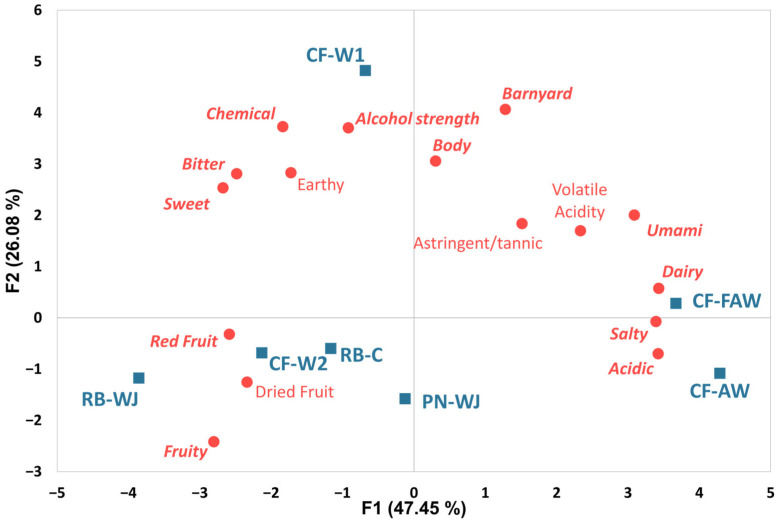
Principal component analysis (PCA) biplot of products (blue) and descriptive analysis attributes (red) on the first two principal components (percentage of total variance explained shown on axes). Bolded, italicized attributes indicate that samples were significantly different in ratings for these attributes as determined by Tukey HSD at a significance level of α = 0.05.

After data collection, one panelist’s data was excluded from the final statistical analysis due to their inability to discriminate between the samples based on the attributes utilized, resulting in a total of 10 panelists included for the final analysis. Mixed-model analysis of variance (ANOVA) was then carried out on each sensory attribute, in which each model included product as a fixed effect, panelist and session as random effects, and all two-way interactions. Tukey HSD means comparisons (α = 0.05) were then generated for each attribute. Across the attributes included in the analysis, significant product effects were observed for barnyard (*p* < 0.0001), chemical (*p* < 0.0001), dairy (*p* < 0.0001), fruity (*p* < 0.0001), red fruit (*p* = 0.002), volatile acidity (*p* = 0.012), acidic (*p* < 0.0001), alcohol strength (*p* = 0.01), bitter (*p* < 0.0001), body (*p* = 0.001), salty (*p* < 0.0001), sweet (*p* = 0.0005), and umami (*p* = 0.001). Dried fruit (*p* = 0.054) and earthy (*p* = 0.058) showed some evidence of a product effect but did not meet the threshold of *p* < 0.05. Astringent/tannic (*p* = 0.633) was not an effective attribute for differentiation of the samples. PCA was conducted using the least-squares means of the sensory attributes for each sample, and a biplot (Figure 1) was constructed with all attributes and samples in two-dimensional space, to comprehensively depict the major trends in the sensory data. In total, the first two principal components accounted for 73.53% of the variance in the data.

Considering the retronasal aroma (Table 3), CF-W1 (7.5) had significantly higher barnyard aroma than the two whey-based piquettes (CF-AW and CF-FAW: 5.4 and 5.0, respectively) and the apple cider-based piquette (RB-C, 4.9).

The water- and juice-based red blend piquette (RB-WJ, 2.8) was significantly lower in barnyard aroma than the previously mentioned samples, while the reference Cabernet Franc-based piquette (CF-W2, 4.7) and the Pinot Noir-based Piquette (PN-WJ, 3.7) were significantly lower in barnyard aroma than CF-W1, but not different from the other two groupings. Chemical aroma was also significantly higher in CF-W1 (6.1) than in all other products, which ranged from 3.5 to 4.6. In the PCA biplot (Figure 1), CF-W1 is quite far from the other products, especially in F2, which is highly associated with barnyard and chemical aromas in the positive direction, indicating that these two attributes served as key characteristics for differentiating CF-W1. Dairy aroma was significantly higher in the two YAW-based samples (CF-AW and CF-FAW: 6.6 and 6.2, respectively) than in all other samples, which ranged from 2.7 to 4.2. This key difference was likely due to the dairy origin of these samples and indicates that carbon filtration was not entirely effective at removing these aromas.

In terms of fruity aroma, RB-WJ (7.7) was significantly higher than all other products, followed by RB-C (5.9), which was significantly higher than all remaining samples aside from CF-W2 (5.6) and PN-WJ (5.4). CF-W2 was significantly higher in fruity aroma than CF-W1 (4.0). The fruity aroma for PN-WJ (5.4) was not significantly higher than any sample. The two whey-based piquettes (CF-AW and CF-FAW: 4.2 and 4.1, respectively) were significantly lower in fruity aroma than RB-WJ and RB-C but were not significantly different from any other product. Red fruit followed a similar trend, with RB-C (6.2) and CF-W2 (6.1) being significantly higher than PN-WJ (4.4) and CF-AW (4.3). Despite a significant product effect for volatile acidity, there were no significant differences between samples using Tukey HSD to compute contrasts, perhaps due to the relatively small range of values for this attribute (3.0–4.4). These trends can be seen in the arrangement of samples in terms of F1 in Figure 1, where dairy was strongly associated with F1 in a positive direction and fruity and red fruit were associated with F1 in a negative direction. This indicates that the whey piquettes were differentiated from the other products by their strong dairy quality and their lower intensity in terms of red fruit and fruity aroma.

Considering the taste and mouthfeel modalities (Table 4), the YAW-based piquettes were significantly more acidic than the other five products (CF-AW and CF-FAW, 9.6 and 9.2, respectively), likely due to increased lactic acid content relative to the other beverages from the production of lactic acid by lactic acid bacteria during the yogurt-making process.

The next highest sample for acidic was RB-C (7.1), likely due to the cider base for this beverage; RB-C was significantly higher in acidity than CF-W2 (5.7) and RB-WJ (5.1), but not significantly higher than CF-W1 (6.0) or PN-WJ (6.7). PN-WJ was rated significantly more acidic than RB-WJ (5.1) but was otherwise not significantly different from the other products aside from the YAW piquettes. CF-W1 was significantly lower in acidity than the YAW products but was otherwise not significantly different from any other product. CF-W2 was significantly lower in acidity than the top three products (CF-AW and FAW, RB-C) but was not significantly different from the remaining products. RB-WJ was significantly lower in acidity than all products aside from CF-W1 and CF-W2.

Alcohol strength spanned a relatively narrow range (3.8–4.7) and was significantly higher in CF-W1 (4.7) than CF-AW (3.8) and PN-WJ (3.8), while the remaining products were not significantly different from each other. CF-W1 (4.9) was significantly more bitter than the two YAW piquettes (both rated 2.7), RB-C (3.0), and PN-WJ (3.4), while RB-WJ (4.4) was only significantly more bitter than the former three (YAW piquettes and RB-C). CF-W2 (3.8) was not significantly different than any other product in terms of bitterness intensity. Ratings for body were significantly higher in CF-FAW (5.1), CF-W1 (5.0), CF-W2 (4.6), and RB-C (4.6) than in PN-WJ (3.4), while the remaining samples were not significantly different from any other sample (CF-AW and RB-WJ at 4.0 and 4.1, respectively). In terms of saltiness, the two YAW-based piquettes (CF-AW and CF-FAW, 5.1 and 4.1, respectively) were rated significantly higher than the remaining products, which ranged from 1.4 to 2.4. This stark difference is to be expected, due to the high mineral content of YAW relative to that of water, grape juice, and wine, but may lead to an undesirable level of saltiness for an alcoholic beverage. Sweetness ratings were on the lower end (0.9–2.0), indicating the relative dryness of the products included, with CF-W1 (2.0) and RB-C (1.8) being rated as significantly sweeter than the two YAW piquettes (CF-AW and CF-FAW, both 0.9), and the remaining products being undifferentiated from these two groupings. Lastly, umami ratings were significantly higher in the two YAW piquettes (CF-AW and CF-FAW, 3.6 and 3.7, respectively) and CF-W1 (3.3) than in RB-WJ (1.9), while the remaining products were not significantly different from these two groups.

These differences are clearly depicted in Figure 1, where F1 is strongly associated with salty, acidic, and umami in the positive direction and with sweet and bitter in the negative direction. In the taste and mouthfeel modalities, F1 effectively separates the whey piquettes (CF-AW and CF-FAW—right hand side) from the other products based on their high acidity, saltiness, and umami, and lower sweetness and bitterness. Taken as a whole, the results of the descriptive analysis show that the whey piquettes had different characteristics than the other products based on their higher dairy aroma and lower fruity and red fruit aroma, in addition to their higher acidity, saltiness, and umami, and their lower bitterness and sweetness. CF-W1 was differentiated from the remaining samples based on its higher barnyard and chemical aromas, and its higher alcohol strength. Among the remaining four samples, the main differences were based on fruity and red fruit aromas and acidity, which differentiated PN-WJ (high in acidity, lower in fruity, red fruit) from RB-WJ. As the primary goal of this study was to characterize piquette and YAW piquette beverages, consumer acceptance testing was not included. Thus, while the YAW beverages received higher ratings for dairy aroma and saltiness, the impact of these attributes on consumer liking and acceptance remains uncertain. To clarify whether these differences drive negative consumer reactions, future research should focus on hedonic testing combined with just about right scaling and penalty analysis [37]. If these attributes prove to be detrimental to consumer acceptance, future research should also assess remediation strategies such as blending the finished product with non-YAW-based product or blending the YAW with other fermentable beverage bases such as grape juice, wort, or water prior to fermentation. Process parameters such as fermentation temperature, yeast pitch rate, yeast strain, pomace variety, pomace pretreatment, and pomace: liquid ratio could also be investigated. The results of this study indicate that the carbon treatment utilized did not fully remove dairy aroma from the YAW, but it is possible that other carbon treatment schema may prove effective.

### 3.2. Chemical Composition of Piquette Beverages

#### 3.2.1. pH, Titratable Acidity, and HPLC Data

As a new and not strictly defined category of beverage, it is unsurprising that the piquettes included in this study varied greatly in their compositions, owing to the differences in production practices including potential additions of water, whey, juice, or other fermentable substrates during production (Table 5). Ethanol content ranged broadly from 4.2 to 8.2% ABV. Between the whey beverages, CF-AW (4.2%) had a lower ABV than CF-FAW (5.8%), which was expected given that the FAW base was supplemented with additional fermentable substrate (dextrose) prior to piquette production. The ABV of the commercial piquette samples largely reflects their varied production practices, with RB-WJ, which was supplemented with the most juice, having the highest ABV (8.2%).

The wide range in ABV among these products presents an interesting opportunity for producers to tune their products to the respective market opportunities that they are seeking to meet. Some producers appear to be aiming for a hard-seltzer-like product, while other products approach more of a sparkling wine. The whey-based piquettes also had the lowest pH values (aside form RB-WJ), reflecting the tartaric acid additions that took place prior to fermentation to bring the products into the typical pH range for wine (3–3.7) [38]. The remaining piquettes had pH values higher than those typical of wines, with most falling between 4 and 5. RB-C had a pH approaching 6, which may be a negative indicator in terms of microbial stability and color stability [38,39].

In terms of sugar utilization, most of the piquettes were fermented to dryness, aside from RB-WJ and PN-WJ, which contained very small quantities of residual glucose and fructose, respectively. The whey piquettes contained small amounts of residual lactose (1.6–2.9 g/L) indicating that the added lactase was not able to completely hydrolyze the lactose during the fermentation process. CF-AW also contained a small amount of residual glucose. Acetic acid was present at low levels in all of the piquettes, likely resulting from the metabolism of heterofermentative lactic acid bacteria (LAB) or yeasts introduced with the grape pomace during production, or of intentionally inoculated or unintentionally introduced malolactic bacteria [40].

Malolactic fermentation (MLF) is a term used to describe the conversion of malic acid to lactic acid via the metabolism of various LAB, and is commonly utilized by winemakers to reduce titratable acidity and sourness perception in wine [41]. To some extent, the higher concentration of lactic acid and absence of malic acid in RB-C may explain its relatively high pH. The remaining commercial piquettes had varying levels of lactic acid and malic acid, which may be indicative of the different levels of MLF conducted by winemakers combined with production factors. The whey piquettes had relatively high levels of lactic acid, which was most likely due to acidification by LAB during yogurt-making as opposed to MLF. Tartaric acid concentrations were lower in the whey beverages, which may indicate a lower proportion of grape or fruit ingredients, since there is very little tartaric acid in YAW [16]. CF-W1 and RB-C were higher in acetic acid, likely because these products were spontaneously fermented and, therefore, had more opportunity for acetic acid producers to access the substrate.

In a recent publication and the first, explicitly on piquette production, Watrelot and Hollis produced piquettes using water, Petite Pearl, or Marquette pomace, and juice using different pomace/water ratios (1:2, 1:2.5, 1:5) and different yeast strains [10]. Across the eight piquettes they produced, pHs ranged from 3.35 to 3.61, TAs ranged from 5.15 to 9.08 g/L tartaric acid-equivalent, %ABVs ranged from 3.63 to 4.77, tartaric acid ranged from 1.00 to 1.55 g/L, and very low quantities of fructose (≤1.93 g/L), lactic acid (≤0.09 g/L), and acetic acid (≤0.21 g/L) were detected. The products in the current study spanned a wider range of compositional profiles, especially in terms of %ABV, demonstrating the wide range of product offerings sold as piquettes.

#### 3.2.2. Aroma Chemistry and Multivariate Analysis

**Figure 2:**
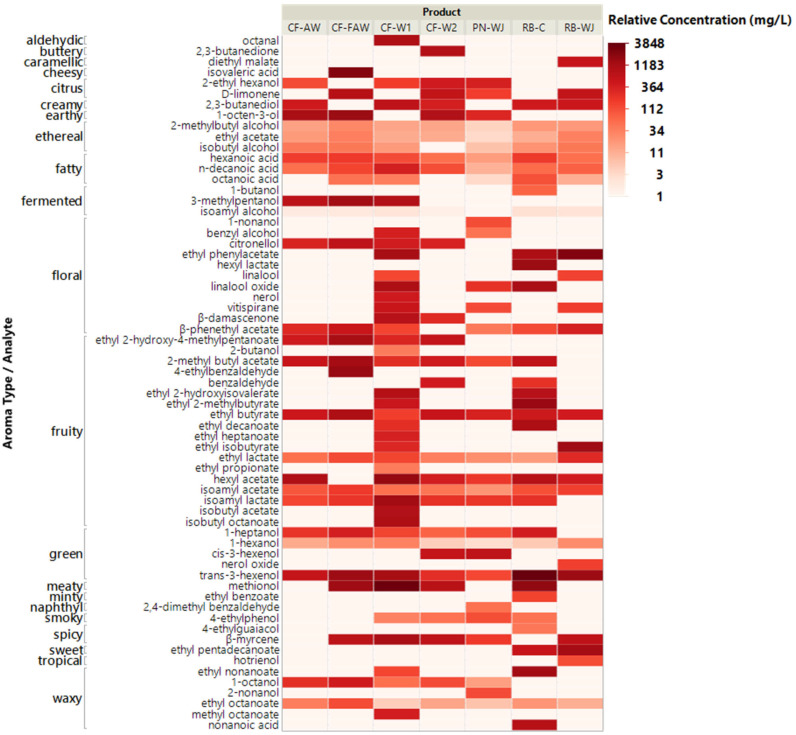
Heatmap of selected aroma compounds identified in piquette products by HS-SPME-GCMS/MS, arranged by flavor type from The Good Scents Database [42].

Headspace Solid-Phase Microextraction–Gas Chromatography–Mass Spectrometry (HS-SPME-GC-MS/MS) analysis of the seven piquette products resulted in the identification of 94 compounds. This list was reduced to 81 after removing compounds that only appeared in one instrumental replicate; were not significantly different across samples based on the instrumental replicates; or were significantly different between instrumental replicates. Of the 81 remaining compounds, 65 had been previously annotated with aroma characteristics in the Good Scents or Flavornet aroma/flavor databases [42,43] (Figure 2, Appendix A), and the remaining 16 were not included in the discussion below (2-propylheptanol; 5-methyl-2-hexanol; 2,4,5-trimethyl-1,3,-dioxolane; 2,2,4-trimethyl-1,3,pentanediol diisobutyrate; 2,3,-di-tertbutylphenol; 2,5-cyclohexadien-1-one; 2-methylbutyl octanoate; 3-isopropyl-1-pentanol; 3-methylbutyl 2-methoxyacetate; 1,3-bis(1,1- dimethylethyl)-benzene; ethyl isopentyl succinate; ethyl succinate; propanoic acid, 2-methyl-, 2,2-dimethyl-1-(2-hydroxy-1-methylethyl)propyl ester; propanoic acid, 2-methyl-,3-hydroxy-2,2,4-trimethylpentyl ester; tris(2-chloropropyl) phosphate).

A wide variety of aroma compounds were identified, including a range of higher alcohols, organic acids, aldehydes, esters, terpenes/terpenoids, and phenolic compounds, in addition to some ketones and one sulfur compound (methionol). In the context of winemaking, each of these compound classes have complex origins that reflect both the raw materials used and their interactions with microbial biochemistry, modulated by processing conditions. Organic acids, their esters, and higher alcohols are generally produced by yeast and/or LAB during fermentation [38,44,45]. Terpenoids are largely grape-derived, and their distribution and quantity is affected by process conditions (extent of skin contact, temperature), potential microbe-derived enzymatic transformations such as enzymatic reduction or hydrolysis of terpenoid glycosides, and chemical transformations [46]. Aldehydes and ketones in wine originate as fermentation metabolites and also through non-enzymatic oxidation pathways [47]. It is additionally worth noting that many detected aroma compounds have been measured in both wine and yogurt, and are likely contributions from yogurt production, pomace, and piquette fermentation in the context of the YAW beverages [48].

The concentrations of these compounds were highly variable across the different products, and ranged from being lower than the limit of detection to a maximum of 3848 mg/L. It is worth reiterating that a semi-quantitative method was used, with a single internal standard. Therefore, the concentrations reported here are not absolute and should be considered relative. In addition, many compounds were identified in multiple samples, while some were present only in one sample. Notably, CF-W1 was higher in many floral and fruity compounds (terpene alcohols, esters, higher alcohols, and terpenoids) than the other samples tested, although this did not necessarily translate to a strong floral/fruity aroma experience in the product. Higher concentrations terpene-based aroma compounds in CF-W1 are likely contributed by the specific pomace used in this product, while fermentation-derived compounds may be attributed to specific microbial contributions, although experimental manipulation of these factors was outside of the scope of this work [41,46].

**Figure 3:**
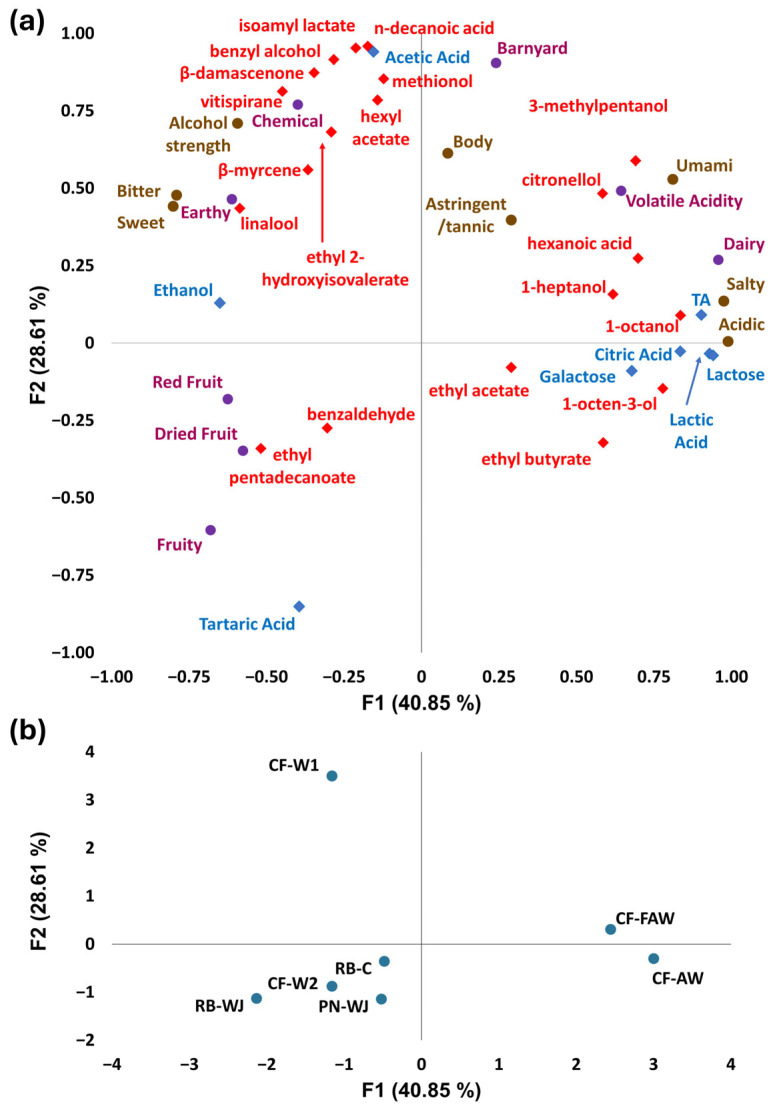
(**a**) correlation map of sensory attributes (taste/mouthfeel—brown circles; retronasal aroma—purple circles), pH, TA, HPLC data (blue diamonds), and selected HS-SPME-GCMS/MS data (red diamonds) on the first two dimensions (percentage of total variance explained shown on axes); (**b**) sample map of piquette products projected in the same space.

To elucidate which specific aroma compounds impacted panelists’ perceptions of the products, a multiple factor analysis was performed. A correlation map and sample map were generated after the MFA, showing the observations and the samples, respectively, both plotted on the first plane (Figure 3). The inputs were the least-squares means for aroma attributes and taste/mouthfeel attributes as two separate groups, in addition to HPLC, pH, and TA as one group, and the GC-MS data as the final group. Groups were evenly weighted and treated as active observations in the analysis. For the GC-MS data, the criteria for inclusion in the MFA were that the analyte was detected in more than one sample and that the analyte’s concentration was significantly correlated with one or more of the sensory attribute scores. The positions of the sensory attributes on the first plane were spatially very similar to the PCA (Figure 1) with dairy, volatile acidity, saltiness, acidity, and umami being associated with positive F1 and red fruit, dried fruit, fruity, chemical, and earthy associated with negative F1. In terms of aroma compounds, 1-octanol (waxy, green, orange, aldehydic, rose, mushroom), 1-octen-3-ol (mushroom, earthy, green, oily, fungal, raw chicken), hexanoic acid (sour, fatty, sweaty, cheesy), 1-heptanol (musty, leafy, violet, herbal, green, sweet, woody, peony), 3-methylpentanol (fusel, cognac, winey, cocoa, green, fruity), citronellol (floral, leathery, waxy, rose, citrus), and ethyl butyrate (fruity, juicy fruit, pineapple, cognac) had the strongest positive association with F1 [42].

The proximity of these volatiles to the dairy and umami attributes indicates that samples that were high in dairy aroma and volatile acidity were also high in these constituents, and that the perception of these aroma qualities may be related to these specific compounds. F1 was also positively associated with TA, lactic acid, citric acid, lactose, and galactose, indicating that sensory acidity and instrumental measures of acidity were closely related. Characteristic aromas of mushrooms and fatty may also have contributed to the perception of umami, although umami is recognized as a taste and not an aroma. Nonetheless, these savory notes may have resulted in inflated umami scores.

Looking in the negative F1 direction, linalool (citrus, floral, sweet, bois de rose, woody, green, blueberry), ethyl pentadecanoate (honey, sweet), and vitispirane (floral, fruity, earthy, woody) [42] had the strongest associations among the aroma compounds, while ethanol content was also strongly negative in F1. This indicates that the fruity and red fruit aromas may have been related to linalool and vitispirane content, while sweet perception may have been affected by increased ethyl pentadecanoate content or higher ethanol content. F2 was mostly characterized by barnyard, chemical, and alcohol strength in the positive direction, and fruity, dried fruit, and red fruit in the negative direction. N-decanoic acid (rancid, sour, fatty, citrus), isoamyl lactate (fruity, creamy, nutty), benzyl alcohol (floral, rose, phenolic, balsamic), β-damascenone (natural, sweet, fruity, rose, plum, grape, raspberry, sugar), methionol (sulfurous, onion, sweet, soup, vegetable), vitispirane, and hexyl acetate (fruity, green, apple, banana, sweet) [42] were correlated with positive F2, indicating that they may have contributed to the barnyard, chemical, or volatile acidity aroma perception of these products; acetic acid was also strongly correlated with positive F2, which is to be expected since it is a key component of volatile acidity. In negative F2, benzaldehyde (sharp, sweet, bitter almond, cherry) and ethyl decanoate (fruity, fatty) [42] are associated with the fruity, red fruit, and dried fruit attributes; tartaric acid is also strongly negative in F2, likely due to CF-W1 being the only sample that did not contain tartaric acid and CF-W1 driving much of the variation in F2.

Considering the sample map, the products tested were generally segregated into three separate regions on the first plane based on their sensory and chemical profiles. The YAW piquettes (CF-AW and CF-FAW) were positive in F1, while all other products were negative on F1, indicating that YAW piquettes were characterized by higher ratings for dairy, salty, acidic, and umami characteristics; higher TA and higher concentrations of lactose, lactic acid, citric acid, galactose, hexanoic acid, 3-methylpentanol, 1-octanol, and 1-octen-3-ol; lower ratings for bitterness, sweetness, red fruit, dried fruit, and fruity; and lower concentrations of ethanol, linalool, and ethyl pentadecanoate. While many of the YAW piquette-associated aroma compounds can be derived from microbial activity in both piquette and yogurt production, higher concentrations of specific yogurt-associated compounds (hexanoic acid, 1-octen-3-ol) may indicate that aroma carryover from yogurt production processes led to pronounced dairy notes in these products [48].

Between CF-AW and CF-FAW, the former was in the negative F2 part of the plane, while the latter was in the positive part of the plain, possibly due to the higher content of n-decanoic acid and isoamyl lactate and lower concentration of tartaric acid in CF-FAW, in spite of the higher ratings for barnyard and higher acetic acid content of CF-AW. CF-W1 was the only product with a large positive F1 on the sample map, driven by its significantly higher barnyard and chemical aromas compared to the other products, and its higher concentrations of n-decanoic acid, isoamyl lactate, benzyl alcohol, β-damascenone, and methionol. Interestingly, despite the higher number and concentration of many fruity aroma compounds in CF-W1, it was perceived as the least fruity sample. This is unsurprising, however, given the complex nature of aroma chemistry of food and beverages, in which complex combinations of different compounds can amplify or mask their individual aroma qualities [49].

The remaining four products are all clustered in quadrant 3, indicating their lower barnyard, chemical, dairy, umami, and acidic qualities compared to CF-W1 and the YAW piquettes, respectively. They were also lower in acetic acid than CF-W1, and lower in titratable acidity and lactic acid than the YAW piquettes, in addition to being lower than CF-W1 and the YAW piquettes in terms of their respective characteristic aroma compounds that were previously described. These products (PN-WJ, RB-C, CF-W2, and RB-WJ) were also generally rated higher in terms of red fruit, dried fruit, and fruitiness, and contained more tartaric acid. Among these four products, RB-WJ distinguished itself with its relatively higher fruitiness, bitterness, and sweetness, all of which correlate with the fact that it had the highest absolute value among the negative F1 samples.

## 4. Conclusions

The aim of this study was to explore and characterize the chemical properties and organoleptic profiles of a subset of products from a new beverage category—piquettes made using grape pomace—and to assess the use of a new raw material, yogurt acid whey, in this product. Overall, the products tested showed a wide range of sensory and compositional profiles, likely driven by different production approaches in terms of ingredients and processing. The whey-based piquettes were quite different from the commercial products, mainly in terms of their acidity, saltiness, and dairy notes, which may limit their acceptability as a part of this beverage category. However, the wide variety of product types and lack of consumer expectations implicit in this new beverage category may allow for the acceptance of such beverages. Consumer acceptance testing was not included in this study, so no definitive conclusions can be drawn as to whether potentially polarizing attributes in the YAW beverages impact consumer liking.

Future research should address this by applying hedonic testing combined with JAR scaling and penalty analysis. Depending on consumer acceptance, blending of the YAW with other matrices such as juice, wort, or water prior to fermentation, or varying yeast variety, pomace variety, pomace, liquid ratio, or fermentation conditions should be explored to mask potentially undesirable tastes/aromas. Future work should also address potential barriers to scale-up including identifying efficient and accessible pasteurization methods for raw materials and assessing the impact raw material transportation has on overall product sustainability. Despite these challenges, piquettes may appeal to consumers who are concerned about the health implications of traditional wine consumption, while simultaneously appealing to consumers who desire products that are novel and contribute to environmental sustainability [50,51]. Piquettes and whey-based piquettes offer a unique opportunity for the beverage industry to upcycle food processing waste into a product that is inexpensive to produce and allows engagement with new consumer segments, and the research presented here can serve as a starting point for understanding potential entry points for new product offerings.

## Figures and Tables

**Table 1:** Production information for yogurt acid whey (CF-AW, CF-FAW) and commercial piquette beverages included in this study.

Sample	Fermentation Media	Pomace Varietal(s)	Origin	Vintage
Cabernet Franc—Acid Whey **(CF-AW)**	Pasteurized, carbon-filtered YAW	Cabernet Franc	New York, US	2022
Cabernet Franc—Fermented Acid Whey **(CF-FAW)**	Fermented YAW drink base			2022
Cabernet Franc—Water 1 **(CF-W1)**	Water			2020
Cabernet Franc—Water 2 **(CF-W2)**	Water, sugar			2022
Red Blend—Cider **(RB-C)**	Apple Cider	Merlot, Cabernet Franc, Gamay		2021
Pinot Noir—Water/Juice **(PN-WJ)**	Water, pinot gris juice	Pinot Noir	Washington, US	2021
Red Blend—Water/Juice **(RB-WJ)**	Water, juice	Blaufrankisch, Riesling, Pinot Noir	Slovenia	2020

**Table 2:** Attributes used in descriptive sensory analysis with corresponding descriptive language and consensus intensity ratings for internal reference sample (CF-W2).

Attribute	Descriptive Language	Consensus Intensity Rating of CF-W2
Retronasal Aroma Attributes		
Red Fruit	Strawberry ^1^, raspberry ^1^	6.5
Fruity	Apple ^1^, pear ^1^, peach ^1^	6
Barnyard	Manure, horse blanket, leather ^1^, musk ^1^	5
Dairy	Greek yogurt, cheese	3
Chemical	Dental filler, solvent	3.3
Volatile acidity	Vinegar, acetone	2.3
Dried Fruit	Apricot ^1^, date, prune ^1^	4.6
Earthy	Mushroom ^1^, dirt, forest floor	3.5
Taste and mouthfeel attributes		
Sweet		1.4
Acidic		6.4
Bitter		3.5
Astringent/tannic		6.4
Alcohol strength		4
Body		4.5
Umami		2.3
Salty		2

^1^ Name of an aroma standard in Le Nez Du Vin set.

**Table 3:** Least-squares means of retronasal aroma attribute intensity ratings of piquette beverages.

Product	Sensory Attribute							
	Barnyard	Chemical	Dairy	Dried Fruit	Earthy	Fruity	Red Fruit	Volatile Acidity
CF-AW	5.4 b	4.1 b	6.6 a	3.2 a	2.5 a	4.2 cd	4.3 b	4.4 a
CF-FAW	5.0 b	3.5 b	6.2 a	3.3 a	3.4 a	4.1 cd	4.7 ab	4.3 a
CF-W1	7.5 a	6.1 a	4.2 b	3.4 a	4.4 a	4.0 d	5.0 ab	4.2 a
CF-W2	4.7 bc	3.5 b	2.7 b	4.3 a	4.1 a	5.6 bc	6.1 a	3.0 a
PN-WJ	3.7 bc	4.2 b	3.4 b	3.4 a	3.6 a	5.4 bcd	4.4 b	3.2 a
RB-C	4.9 b	4.1 b	3.7 b	4.7 a	2.9 a	5.9 b	6.2 a	4.4 a
RB-WJ	2.8 c	4.6 b	2.4 b	4.0 a	3.5 a	7.7 a	5.7 ab	3.2 a

Means within a column that share the same letter are not significantly different, as determined by Tukey HSD with α = 0.05.

**Table 4:** Least-squares means of taste and mouthfeel attribute intensity ratings of piquette beverages.

Product	Taste or Mouthfeel Attribute							
	Acidic	Alcohol Strength	Astringent/ Tannic	Bitter	Body	Salty	Sweet	Umami
CF-AW	9.6 a	3.8 b	6.5 a	2.7 c	4.0 ab	5.1 a	0.9 b	3.6 a
CF-FAW	9.2 a	4.0 ab	7.3 a	2.7 c	5.1 a	4.1 a	0.9 b	3.7 a
CF-W1	6.0 bcd	4.7 a	7.0 a	4.9 a	5.0 a	2.4 b	2.0 a	3.3 a
CF-W2	5.7 cd	4.0 ab	6.7 a	3.8 abc	4.6 a	1.8 b	1.4 ab	2.7 ab
PN-WJ	6.7 bc	3.8 b	7.0 a	3.4 bc	3.4 b	2.0 b	1.1 ab	2.4 ab
RB-C	7.1 b	4.2 ab	6.6 a	3.0 c	4.6 a	2.4 b	1.8 a	2.4 ab
RB-WJ	5.1 d	4.3 ab	6.4 a	4.4 ab	4.1 ab	1.4 b	1.6 ab	1.9 b

Means within a column that share the same letter are not significantly different as determined by Tukey HSD with α = 0.05.

**Table 5:** pH and concentrations of sugars, organic acids, titratable acidity (TA), and ethanol of piquette beverages.

Sample Code	CF-AW	CF-FAW	CF-W1	CF-W2	PN-WJ	R-WJ	RB-C
Ethanol (% abv)	4.2	5.8	6.6	6.2	4.2	8.2	6.6
pH	3.43	3.50	4.47	4.01	3.54	3.34	5.95
TA (g/L tartaric acid)	8.5	7.6	4.4	2.9	3.4	4.3	5.1
Glucose (g/L)	0.4	n.d.	n.d.	n.d.	n.d.	0.3	n.d.
Fructose (g/L)	n.d.	n.d.	n.d.	n.d.	0.2	n.d.	n.d.
Lactose (g/L)	2.9	1.6	n.d.	n.d.	n.d.	n.d.	n.d.
Galactose (g/L)	8.0	n.d.	n.d.	n.d.	n.d.	n.d.	0.7
Acetic Acid (g/L)	0.95	0.77	3.08	0.58	0.90	0.48	1.28
Malic Acid (g/L)	0.5	1.2	n.d.	n.d.	n.d.	2.1	n.d.
Tartaric Acid (g/L)	0.7	0.6	n.d.	1.8	2.0	1.5	1.0
Citric Acid (g/L)	1.8	1.3	n.d.	n.d.	n.d.	0.1	n.d.
Lactic Acid (g/L)	5.3	3.5	1.2	1.9	0.8	0.2	3.9

n.d. indicates that the analyte was not detected during HPLC-RID.

## Data Availability

The data presented in this study is available on request from the corresponding author.

## Supplementary Materials

The following supporting information can be downloaded at https://www.mdpi.com/article/10.3390/foods14183240/s1, Table S1: Detailed information of 100 analytical standard compounds and 1 internal standard (highlighted in green) used for the development and quality assurance of the HS-SPME-GC-MS/MS method.

## Appendix A

**Table A1:** Aroma compounds with aroma types and descriptors from the Good Scents and Flavornet databases.

Chemical	Good Scents Aroma Type [42]	Good Scents Aroma Description [42]	Flavornet Aroma Description [43]
octanal	aldehydic	aldehydic, waxy, citrus, orange peel, green, herbal, fresh, fatty	fat, soap, lemon, green
2,3-butanedione	buttery	buttery, sweet, creamy, pungent, carmellic	butter
2-methyl butyl acetate	fruity	fruit, overripe fruit, sweet, banana, juicy fruit, fruity	
diethyl malate	caramellic	caramellic, sugar, brown sugar, sweet, winey, fruity, herbal	
isovaleric acid	cheesy	sour, sweaty, cheesy, tropical	sweat, acid, rancid
2-ethyl hexanol	citrus	citrus, fresh, floral, oily, sweet	
D-limonene	citrus	citrus, orange, fresh, sweet	citrus, mint, lemon, orange
2,3-butanediol	creamy	fruity, creamy, buttery	fruit, onion
1-octen-3-ol	earthy	mushroom, earthy, green, oily, fungal, raw chicken	
2-methylbutyl alcohol	ethereal	ethereal, fusel, alcoholic, fatty, greasy, winey, whiskey, leathery, cocoa	
ethyl acetate	ethereal	ethereal, fruity, sweet, weedy, green	pineapple
isobutyl alcohol	ethereal	ethereal, winey, fusel, whiskey	wine, solvent, bitter
hexanoic acid	fatty	sour, fatty, sweaty, cheesy	
n-decanoic acid	fatty	rancid, sour, fatty, citrus	rancid, fat
octanoic acid	fatty	fatty, waxy, rancid, oily, vegetable, cheesy	sweat, cheese
3-methylpentanol	fermented	fusel, cognac, winey, cocoa, green, fruity	
1-butanol	fermented	fusel, oily, sweet, balsamic, whiskey	medicine, fruit
isoamyl alcohol	fermented	fusel, alcoholic, whiskey, fruity, banana	whiskey, malt, burnt
1-nonanol	floral	fresh, clean, fatty, floral, rose, orange, dusty, wet, oily	fat, green
benzyl alcohol	floral	floral, rose, phenolic, balsamic	slightly sweet floral, damp-wet/sweet, flower
citronellol	floral	floral, leathery, waxy, rose, citrus	rose
ethyl phenylacetate	floral	sweet, floral, honey, rose, balsamic, cocoa	fruit, sweet
hexyl lactate	floral	sweet, floral, green, fruity	
linalool	floral	citrus, floral, sweet, bois de rose, woody, green, blueberry	flower, lavender
linalool oxide	floral	floral, herbal, earthy, green	flower, wood
nerol	floral	sweet, natural, neroli, citrus, magnolia	sweet
vitispirane	floral	floral, fruity, earthy, woody	
β-damascenone	floral	natural, sweet, fruity, rose, plum, grape, raspberry, sugar	apple, rose, honey
β-phenethyl acetate	floral	floral, rose, sweet, honey, fruity, tropical	rose, honey, tobacco
2-butanol	fruity	fruity, sweet apricot	
4-ethylbenzaldehyde	fruity	almond, bitter almond, sweet, anise, cherry	sweet
benzaldehyde	fruity	sharp, sweet, bitter almond, cherry	almond, burnt sugar
ethyl 2-hydroxyisovalerate	fruity	pineapple, strawberry, tea, honey	
ethyl 2-methylbutyrate	fruity	sharp, sweet, green, apple, fruity	apple
ethyl decanoate	fruity	fruity, fatty	grape
ethyl butyrate	fruity	fruity, juicey fruit, pineapple, cognac	apple
ethyl heptanoate	fruity	fruity, pineapple, cognac, rummy, winey	
ethyl isobutyrate	fruity	sweet, ethereal, fruity, alcoholic, fusel, rummy	sweet, rubber
ethyl lactate	fruity	sharp, tart, fruity, buttery, butterscotch	fruity
ethyl propionate	fruity	sweet, fruity, rum, juicy, fruit, grape, pineapple	fruit
hexyl acetate	fruity	fruity, green, apple, banana, sweet	fruit, herb
isoamyl lactate	fruity	fruity, creamy, nutty	
isobutyl acetate	fruity	sweet, fruity, ethereal, banana, tropical	fruit, apple, banana
isobutyl octanoate	fruity	fruity, green, oily, floral	
1-heptanol	green	musty, leafy, violet, herbal, green, sweet, woody, peony	herb, mushroom, chemical, green
1-hexanol	green	green, fruity, apple skin, oily	resin, flower, green
trans-3-hexenol	green	green, cortex, privet, leafy, floral, petal, oily, earthy	
cis-3-Hexenol	green	fresh, green, grassy, foliage, vegetable, herbal, oily	
nerol oxide	green	green, weedy, cortex, herbal, narcissus, celery	oil, flower
isoamyl acetate	fruity	sweet, fruity, banana, solvent	banana
methionol	meaty	sulfurous, onion, sweet, soup, vegetable	sweet, potato
ethyl benzoate	minty	fruity, dry, musty, sweet, wintergreen	chamomile, flower, celery, fruit
ethyl 2-hydroxy-4-methylpentanoate	fruity	fresh blackberry	
2,4-dimethyl benzaldehyde	naphthyl	naphthyl, cherry, almond, spicy, vanilla	
4-ethylphenol	smoky	phenolic, castoreum, smoky, guaiacol	must
4-ethylguaiacol	spicy	spicy, smoky, bacon, phenolic, clove	
β-myrcene	spicy	peppery, terpenic, spicy, balsamic, plastic	balsamic, must, spice
ethyl pentadecanoate	sweet	honey, sweet	
hotrienol	tropical	sweet, tropical, floral, fennel, ginger, spicy	hyacinth
ethyl nonanoate	waxy	fruity, rose, waxy, rummy, winey, natural, tropical	
1-octanol	waxy	waxy, green, orange, aldehydic, rose, mushroom	moss, nut, mushroom, chemical, metal, burnt
2-nonanol	waxy	waxy, green, creamy, citrus, orange, cheesy, fruity	cucumber
ethyl octanoate	waxy	fruity, winey, waxy, sweet, apricot, banana, brandy, pear	fruit, fat
methyl octanoate	waxy	waxy, green, sweet, orange, aldehydic, vegetable, herbal	waxy type odor/orange
nonanoic acid	waxy	waxy, dirty, cheesy, dairy	green, fat
