# Supplementary material for: Sensory and Chemical Characterization of Upcycled Pomace- and Whey-Based Piquette Beverages
Source: Foods. 2025 Sep 18;14(18):3240. doi: 10.3390/foods14183240 (PMC12470025; doi:10.3390/foods14183240)
Supplement: Supplementary file 1 [file foods-14-03240-s001.zip › foods-3866088-supplementary.pdf]

Table S1 **Detailed information of 100 analytical standard compounds and 1 internal st**

S.NO	Compound name	Supplier
1	Hexanal-d12	CDN Isotopes
2	Propanal, 2-methyl-	Sigma-Aldrich
3	Diacetal	Sigma-Aldrich
4	Methyl propionate	Sigma-Aldrich
5	1,4-Pentadien-3-ol	Sigma-Aldrich
6	Pentanal	Sigma-Aldrich
7	Ethyl propionate	Sigma-Aldrich
8	Methyl butyrate	Thermo Scientific Chemicals
9	3-Buten-1-ol, 3-methyl-	Sigma-Aldrich
10	1-Butanol, 3-methyl-	Sigma-Aldrich
11	1-Butanol, 2-methyl-	Sigma-Aldrich
12	Isobutyl acetate	Sigma-Aldrich
13	Methyl isovalerate	Alfa Aesar
14	Hexanal	Sigma-Aldrich
15	2-Hexanol	Sigma-Aldrich
16	Ethyl butyrate	Sigma-Aldrich
17	propyl propionate	Sigma-Aldrich
18	butyl acetate	TCI
19	2 Methyl pyrazine	Sigma-Aldrich
20	Furfural	Sigma-Aldrich
21	4-methyl-1-pentanol	Sigma-Aldrich
22	isopropyl butyrate	Sigma-Aldrich
23	2-Butenoic acid, ethyl ester	Sigma-Aldrich
24	(E)-3-Hexen-1-ol	Sigma-Aldrich
25	Ethyl 3-methylbutanoate	Sigma-Aldrich
26	2-hexanal	Sigma-Aldrich
27	(Z)-3-Hexen-1-ol	Sigma-Aldrich
28	1-Hexanol	Sigma-Aldrich
29	Heptanal	Sigma-Aldrich
30	Propanal, 3-(methylthio)-	Sigma-Aldrich
31	2-Ethylpyrazine	Sigma-Aldrich
32	Pyrazine, 2,3-dimethyl-	Sigma-Aldrich
33	3-Methyl-2-buten-1-ol acetate	Sigma-Aldrich
34	Methyl hexanoate	Sigma-Aldrich
35	alpha-Pinene	Sigma-Aldrich
36	3-Heptanol, 3-methyl-	Sigma-Aldrich
37	Ethyl 3-hydroxy-Butanoate	Sigma-Aldrich
38	2-Pentanone, 4-mercapto-4-methyl-	Sigma-Aldrich
39	1-Heptanol	Sigma-Aldrich
40	.beta.-Pinene	Sigma-Aldrich
41	Hexanoic acid	Sigma-Aldrich
42	.beta.-Myrcene	Sigma-Aldrich

43	Thiazole, 2,4,5-trimethyl-	Sigma-Aldrich
44	2-Furanmethanol, acetate	Sigma-Aldrich
45	Hexanoic acid, ethyl ester	Sigma-Aldrich
46	Pyrazine, 2-ethyl-3-methyl-	Sigma-Aldrich
47	Pyrazine, trimethyl-	Sigma-Aldrich
48	Octanal	Sigma-Aldrich
49	Acetic acid, hexyl ester	Sigma-Aldrich
50	.alpha.-Phellandrene	Penta International Corporation
51	.alpha.-Terpinene	Sigma-Aldrich
52	p-Cymene	Sigma-Aldrich
53	D-Limonene	Sigma-Aldrich
54	Eucalyptol	Sigma-Aldrich
55	.beta.-(E)-Ocimene	Sigma-Aldrich
56	Acetophenone	Sigma-Aldrich
57	trans,trans-Hexa-2,4-dienyl acetate	Sigma-Aldrich
58	1-Octanol	Sigma-Aldrich
59	2,3-Diethylpyrazine	Sigma-Aldrich
60	trans-Linalool oxide	Sigma-Aldrich
61	Terpinolene	Sigma-Aldrich
62	Linalool	Sigma-Aldrich
63	2(5H)-Furanone, 3-hydroxy-4,5-dimetl	Sigma-Aldrich
64	Nonanal	Sigma-Aldrich
65	Methyl octanoate	Sigma-Aldrich
66	3-Mercaptohexanol	Sigma-Aldrich
67	5-Methyl-6,7-dihydro-5H-cyclopenta[b	Sigma-Aldrich
68	Acetic acid, 2-ethylhexyl ester	Sigma-Aldrich
69	3-Nonen-1-ol, (Z)-	TCI
70	Pyrazine, 2,3-diethyl-5-methyl-	Sigma-Aldrich
71	Citronellal	Sigma-Aldrich
72	2-Nonenal, (E)-	Sigma-Aldrich
73	endo-Borneol	TCI
74	Benzoic acid, ethyl ester	Sigma-Aldrich
75	5-Ethyl-4-hydroxy-2-methyl-3(2H)-fura	Sigma-Aldrich
76	dl-Menthol	Sigma-Aldrich
77	1-Nonanol	Sigma-Aldrich
78	(+-)-Dihydrocarveol	Sigma-Aldrich
79	alpha-Terpineol	Sigma-Aldrich
80	Hexyl butyrate	TCI
81	Ethyl octanoate	Sigma-Aldrich
82	(-)-Myrtenol	Sigma-Aldrich
83	Decanal	Sigma-Aldrich
84	beta-Citronellol	Sigma-Aldrich
85	Hexyl 2-methylbutyrate	Sigma-Aldrich
86	alpha-Citral	Sigma-Aldrich
87	Hexyl 3-methylbutyrate	Sigma-Aldrich

88	(-)-Carvone	Sigma-Aldrich
89	Hexyl acetate	Sigma-Aldrich
90	Linalyl acetate	Sigma-Aldrich
91	Geraniol	Sigma-Aldrich
92	1-Decanol	Sigma-Aldrich
93	(-)-Bornyl acetate	Sigma-Aldrich
94	Carvacrol	Sigma-Aldrich
95	2,4-Decadienal, (E,E)-	Sigma-Aldrich
96	Eugenol	Sigma-Aldrich
97	Hexyl hexanoate	Sigma-Aldrich
98	Caryophyllene	Sigma-Aldrich
99	(Z)-.beta.-Farnesene	Sigma-Aldrich
100	.alpha.-Humulene	Sigma-Aldrich
101	(E)-.beta.-Farnesene	Sigma-Aldrich

tandard (highlighted in green) used for the development and quality assurance of the HS-SPM

Perecent Purity	CAS Number	Chemical Structure
96%	1219803-74-3	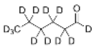
98%	78-84-2	
95%	431-03-8	
99%	554-12-1	
99%	922-65-6	
97%	110-62-3	
99%	105-37-3	
99%	623-42-7	
97%	763-32-6	
99%	123-51-3	
98%	137-32-6	
99%	110-19-0	
98%	556-24-1	
98%	66-25-1	
98%	626-93-7	
99%	105-54-4	
99%	106-36-5	
99%	123-86-4	
99%	109-08-0	
98%	98-01-1	
97%	626-89-1	
99%	638-11-9	
98%	6776-19-8	
99%	928-97-2	
98%	108-64-5	
99%	6728-26-3	
97%	928-96-1	
98%	111-27-3	
98%	111-71-7	
98%	3268-49-3	
98%	13925-00-3	
99%	5910-89-4	
98%	1191-16-8	
99%	106-70-7	
98%	80-56-8	
99%	5582-82-1	
98%	56816-01-4	
99%	19872-52-7	
99%	53535-33-4	
99%	127-91-3	
99%	142-62-1	
95%	123-35-3	

97%	13623-11-5
99%	623-17-6
99%	123-66-0
96%	15707-23-0
99%	14667-55-1
99%	124-13-0
98%	142-92-7
99%	99-83-2
89%	99-86-5
99%	99-87-6
98%	5989-27-5
99%	470-82-6
90%	13877-91-3
98%	98-86-2
97%	1516-17-2
99%	111-87-5
99%	15707-24-1
97%	34995-77-2
90%	586-62-9
97%	78-70-6
97%	28664-35-9
98%	124-19-6
99%	111-11-5
96%	51755-83-0
97%	23747-48-0
99%	103-09-3
99%	10340-23-5
99%	18138-04-0
99%	106-23-0
98%	18829-56-6
95%	464-45-9
99%	93-89-0
96%	27538-09-6
97%	89-78-1
98%	143-08-8
97%	38049-26-2
99%	98-55-5
99%	2639-63-6
99%	106-32-1
95%	515-00-4
99%	112-31-2
96%	106-22-9
95%	10032-15-2
98%	106-26-3
99%	10032-13-0

96%	2244-16-8
98%	136954-20-6
99%	115-95-7
98%	106-24-1
97%	112-30-1
97%	5655-61-8
98%	499-75-2
95%	25152-84-5
99%	97-53-0
97%	6378-65-0
80%	87-44-5
98%	28973-97-9
96%	6753-98-6
98%	18794-84-8

ME-GC-MS/MS method
